# Posting With Purpose: A Strategic Instagram Workflow for Plastic Surgeons

**DOI:** 10.1093/asjof/ojag095

**Published:** 2026-05-19

**Authors:** Nada E Botros, Nicholas Mosca, Sakar Gupta, Ferris Zeitouni, Peter Wirth, Venkat K Rao

## Abstract

Instagram (Meta, Menlo Park, CA) has become an important tool for patient education, practice marketing, and professional branding within plastic surgery. As public interest in aesthetic surgery continues to rise alongside increasing surgeon engagement with visually driven social media, there is a growing need for a practical, ethically grounded framework to guide professional use of Instagram. This article provides a structured, evidence-informed approach to the effective and responsible use of Instagram by plastic surgeons. Key topics discussed include profile optimization, content development strategy, performance analytics, and mitigation of legal and ethical risk. Specific recommendations are provided for educational, marketing, and personal content, with emphasis on maintaining professionalism, transparency, and patient confidentiality. Considerations regarding content timing and posting frequency to optimize audience engagement are also reviewed. When applied strategically, Instagram can serve as a powerful tool for patient education, trust building, and practice growth, while simultaneously facilitating professional networking and knowledge dissemination within the field of plastic surgery.

Social media has become a central extension of plastic surgery clinical practice, professional branding, and patient education. It has fundamentally reshaped how surgeons engage patients and present their professional identity.

Since its release on April 1, 2012, Instagram (Meta, Menlo Park, CA) has undergone tremendous global growth, now boasting over 2 billion monthly active users worldwide.^1^ Its emphasis on image- and video-based storytelling uniquely aligns with the visual nature of plastic surgery, positioning Instagram as a particularly influential platform within the specialty. This expanded adoption of social media has been mirrored within the plastic surgery community. Early in the last decade, social media use among plastic surgeons was limited, with about 30% reporting use in 2010, and only 28.2% actively using social media to advertise their work.^2,3^ By 2020, over 90% of surgeons adopted the use of social media as a tool for patient engagement, professional visibility, and practice marketing.^2^

The profound growth in professional adoption of Instagram among plastic surgeons has coincided with a substantial rise in public interest in plastic surgery, as evidenced by Google Trends data revealing a concurrent increase in search volumes for plastic surgery procedures following Instagram's launch.^4^ Together, these parallel trends underscore Instagram's relevance not simply as a marketing platform but as a critical interface between surgeons and the public. This review provides a practical, surgeon-focused guide to using Instagram effectively and ethically, offering evidence-based strategies for integrating social media into professional practice while preserving clinical integrity and patient trust.

## INSTAGRAM USE IN PLASTIC SURGERY

Plastic surgeons primarily use Instagram for 3 core functions: patient education, marketing, and professional development.^5^ These functions collectively shape how surgeons are perceived by patients, peers, and the broader public.

### Patient Education

Instagram's emphasis on visual content makes it uniquely suited for patient education in plastic surgery. Unlike text-based platforms, Instagram allows surgeons to communicate complex surgical concepts efficiently through images and short-form video, facilitating patient understanding of procedures, risks, recovery, and outcomes.^5^ As patient utilization of Instagram for education and surgeon selection continues to grow, the platform increasingly serves as an entry point through which patients form expectations and make informed decisions regarding care.^6^

### Marketing and Patient Acquisition

The same visual framework that supports education also underpins Instagram's efficacy as a marketing tool. By showcasing surgical outcomes and procedural explanations, surgeons can present their work in an accessible and engaging format that aligns with patient preferences. Empirical evidence supports these advantages, with studies demonstrating measurable increases in patient volume attributable to effective Instagram use.^2^ In addition, posting patterns and engagement metrics provide surgeons with real-time insight into patient interests and demand trends, allowing them to anticipate shifts in procedural interest and tailor practice offerings accordingly.^7^

### Professional Development

Beyond patient-facing applications, Instagram has emerged as an important platform for professional identity development. Plastic surgeons use Instagram to connect with peers, pursue mentorship, and shape public and professional perceptions of the specialty. Instagram's visual format facilitates the dissemination of surgical techniques, case results, and scholarly discussion, fostering informal mentorship that is particularly valuable for trainees and early-career surgeons.^8^ Studies have demonstrated that posts highlighting the surgical team—including behind-the-scenes images and staff introductions—also contribute to professional identity building, fostering relatability and trust with viewers, consistently outperforming text-only posts.^9,10^ Such content reinforces transparency, practice culture, and approachability without compromising professionalism.

However, adoption varies by career stage. Younger and private-practice surgeons are more likely to engage with social media and view it positively, whereas senior and academic surgeons are less likely to participate, often citing concerns related to time investment, professionalism, and privacy.^5,8^ This hesitancy may result in missed opportunities for mentorship, collaboration, and visibility, and can limit the influence of senior surgeons in shaping professional discourse and public understanding of plastic surgery.^5^ Without their active participation, expert voices may be underrepresented in online spaces where patients increasingly seek information.

For surgeons at all career stages, a structured and intentional Instagram presence can therefore function not only as a marketing tool but also as a deliberate extension of professional leadership, mentorship, and patient education.

## UNDERSTANDING INSTAGRAM

To use Instagram effectively as a professional tool, plastic surgeons must understand the platform's core mechanics, including content distribution, audience behavior, and engagement features. Additionally, there are multiple post format types, each with unique benefits and applications, reviewed in Table 1. Instagram operates through proprietary algorithms that determine which content appears in users’ feeds based on factors such as demographic characteristics, geographic location, and prior interaction history.^6^ These systems influence visibility and discoverability, shaping which users encounter a surgeon's content and how often.

**Table 1.** Summary of Instagram Content Formats and Practical Applications in Plastic Surgery

Content type	Primary objective	Utility in plastic surgery	Key strengths	Best practice tips
Image posts	Brand presence; aesthetic showcase	Before-and-after images; infographics and branding	Visually concise; long-term profile visibility long-term; discoverable via hashtag	Add location tag to each post; accompany posts with captions that further provide insight into the procedure, recovery, etc.
Reels (short-form video)	Trend-adapted content; maximized discovery and reach	Educational videos; patient testimonials; behind-the-scenes insights	Most powerful discovery and engagement tool; reaches both followers and nonfollowers	Leverage audio and video-format trends to optimize reach; minimize on-screen text
Stories (24-hour posts)	Real-time updates; direct user engagement	Polls, Q&A stickers, and direct links facilitate user engagement; brief daily updates; upcoming service offerings or events	Boost engagement through interactive features (polls, questions, links, etc.); fosters perceived authenticity; low production barrier	Stories can be archived on a creator's profile for continued viewing using the highlights feature
Live videos	Real-time education and interaction; longer-format discussion	Live Q&A sessions; educational sessions; literature discussions	Useful format to build trust and transparency; promotes direct audience participation	Sessions can be co-hosted with colleagues; recorded live-video sessions can be posted on a creator's profile for future viewing

Overview of Instagram content formats, primary objectives, and best-practice applications for plastic surgeons, highlighting how images, Reels, Stories, and Live videos can be leveraged for education, engagement, and practice visibility.

### Content Distribution and Algorithms

Instagram's algorithm prioritizes content that generates sustained user engagement, including likes, comments, shares, saves, and watch time. Posts that generate strong initial engagement are more likely to be distributed to broader audiences, including nonfollowers.^11^ For plastic surgeons, this means that educational clarity, visual quality, and relevance to patient interests directly influence reach.

In recent years, Instagram has placed increased emphasis on short-form video, particularly through Reels, which are distributed beyond a creator's existing follower base and represent one of the most effective mechanisms for account growth and discovery. For plastic surgeons, Reels provide a high-yield format for brief educational explanations, procedural overviews, recovery timelines, and practice messaging delivered in a concise, visually engaging manner.

### Audience Behavior and Platform Demographics

Effective Instagram use requires recognition that audience behavior varies by age group and platform preference. Younger users, generally under 35 years of age, gravitate toward visually driven platforms such as Instagram, TikTok, and Snapchat, with short-form video demonstrating the highest engagement.^2,6^ These users are more likely to consume content that is concise, visually compelling, and easily shareable.

In contrast, users aged 35 and older tend to engage more frequently with Facebook and YouTube, where longer-form educational content and detailed explanations are preferred.^2^ Because Instagram is integrated with Facebook, plastic surgeons can cross-post content and leverage Facebook's advanced targeting capabilities to reach both younger and older patient populations using a unified content strategy.^6^

### Engagement and Communication Features

Instagram's interactive features—including comments, direct messages (“DMs”), live videos, and stories—enable direct, low-barrier communication between surgeons and the public. When managed appropriately, these tools can enhance accessibility, transparency, and patient trust. However, engagement must remain professional and clearly educational in nature to avoid the perception of individualized medical advice.

From a strategic perspective, engagement metrics also provide indirect feedback on patient interests and emerging demand. Patterns in post performance, saves, and audience interaction offer insight into which topics resonate most strongly with viewers, informing future educational content and outreach efforts. When interpreted thoughtfully, these data allow surgeons to align messaging with patient needs while maintaining ethical and professional boundaries.

## BUILDING AN EFFECTIVE INSTAGRAM PRESENCE

### Creating an Effective Instagram Profile

A professional profile serves as the digital front door to a plastic surgeon's practice, shaping patients’ first impressions.^12^ Profile pictures should be professional headshots, ideally in medical or surgical attire. One study suggests that formal attire, particularly a white coat, is associated with higher perceived professionalism, competence, and trustworthiness, especially among younger surgeons establishing credibility.^12^

Plastic surgeons should use a Business Profile or Creator Profile, which provides access to analytics, advertising tools, and streamlined communication features.^13^ The biography should clearly list specialty, degrees, and board certification status—factors consistently ranked among the most important considerations for surgeon selection.^14^ Contact information, including website links, phone number, email, and location, should be current and actively monitored.^13,15^ Direct messaging and comments should remain enabled, as prompt, professional responses reinforce trust and facilitate conversion to in-person consultation.^13^

## CONTENT STRATEGY

### Educational Content

Educational content forms the foundation of a responsible Instagram presence. Informative posts provide accessible, accurate explanations of procedures, risks, benefits, recovery timelines, and postoperative care, improving patient understanding and preparedness.^6^ One recent study showed that patients consistently place the greatest value on educational and outcome-focused content, which strongly influences both surgeon selection and treatment commitment.^6^

Clear descriptions of procedures and aftercare through Instagram are essential for establishing realistic expectations and minimizing misinformation.^16,17^ Transparent discussion of risks and recovery has been associated with reduced anxiety, improved adherence, and higher patient satisfaction.^16,17^ Educational posts should consistently emphasize patient safety and individualized care, while avoiding patient-specific medical advice. Explicit disclaimers clarifying that information being provided is general, educational content and *not* personalized medical advice or diagnostics are essential to protect both patients and surgeons.

### Before and After Photos

Before-and-after images remain one of the most influential content types in plastic surgery social media.^6,18^ These images allow prospective patients to evaluate outcomes directly and are a primary driver of engagement and surgeon selection.^6,18^

Trust depends on authenticity. Standardized, high-quality photographs that accurately represent outcomes are essential for maintaining credibility.^19,20^ A 2023 study found that 70.8% of before-and-after images from top plastic surgery Instagram accounts demonstrated bias toward the postoperative image, presenting a more flattering result through the use of more optimal angles, lighting, backgrounds, and poses.^19^ While such strategies may produce impressive-appearing results, they ultimately mislead viewers, compromising ethical standards of professional social media use and jeopardizing patient-provider trust.^19,20^ Photos should instead showcase consistency and reality to provide patients with realistic expectations.^21^

Compliance with platform content policies is also critical. Instagram restricts nudity, with limited exceptions (eg, post-mastectomy scarring).^19,22^ Many surgeons mitigate removal risk by posting images with undergarments or branded garments, which simultaneously reinforce brand identity and prevent unauthorized image reuse.^15,23^

Algorithmic down-ranking of body-related content, often referred to as “shadowbanning,” has become a widespread concern. Survey data from 2025 indicate that 68% of plastic surgeons believe their content has been restricted despite guideline compliance.^24^ For surgeons, this means that even compliant, patient-focused content may be less discoverable, limiting its educational value and reducing its effectiveness as a tool for patient acquisition and informed decision-making.^24^ Although Instagram does not formally recognize shadowbanning, it acknowledges limiting the recommendation of content deemed sensitive, meaning posts may remain visible to existing followers but are less likely to appear in discovery pathways such as Explore, hashtag feeds, or nonfollower recommendations.^25^ Regular review of Account Status and adherence to Recommendation Guidelines may help identify potential guideline violations and mitigate reduced visibility.

### Personal/Lifestyle Posts

Personal or lifestyle content can foster relatability and trust with potential patients; however, its impact is nuanced and must be balanced with professionalism and educational value. A study examining the top 10 global plastic surgeons on Instagram revealed a notable paradox in content engagement: although marketing-focused posts constituted the majority of content (64.5%), personal (20%) and educational (4.5%) posts generated disproportionately higher levels of engagement.^4^ Prior studies suggest that physicians’ professional and educational social media activity is associated with patient adherence, whereas predominantly personal content may be associated with lower adherence.^26^ Maintaining a clear distinction between professional and personal content is therefore important for sustaining engagement while preserving patient trust.

### Marketing Posts

Despite representing the greatest proportion of content among plastic surgeons, marketing posts consistently generate the lowest levels of engagement, with users instead favoring before-and-after images, testimonials, and informative content.^6,7^

Nonetheless, marketing posts remain an important component of an effective Instagram strategy when used intentionally. Instagram's promotional features enable targeted advertising that extends beyond organic reach.^13,15^ Surgeons can specify geographic location, demographic characteristics (eg, age, gender, interests), and desired engagement outcomes such as profile visits, website clicks, or follows.^13,15^ These tools allow plastic surgeons to reach local patient populations or focus on groups most likely to pursue specific procedures. Instagram also provides detailed performance analytics, including reach, impressions, engagement, click-through rates, and cost-per-result, enabling data-driven optimization of marketing campaigns.^13,15^ Surgeons should use these metrics to evaluate effectiveness and refine strategy over time.

All marketing content must strictly comply with the advertising standards set forth by organizations such as the American Society of Plastic Surgeons (ASPS) and the American Board of Plastic Surgery (ABPS).^22,27,28^ These standards demand that posts be accurate, not deceptive, and honor patient confidentiality and dignity.^22,27,28^ Surgeons should steer clear of exaggerated claims, sensationalized messaging, and inadequate disclosure of credentials.^22,27,28^

### Frequency and Timing of Posts

**Figure 1.**
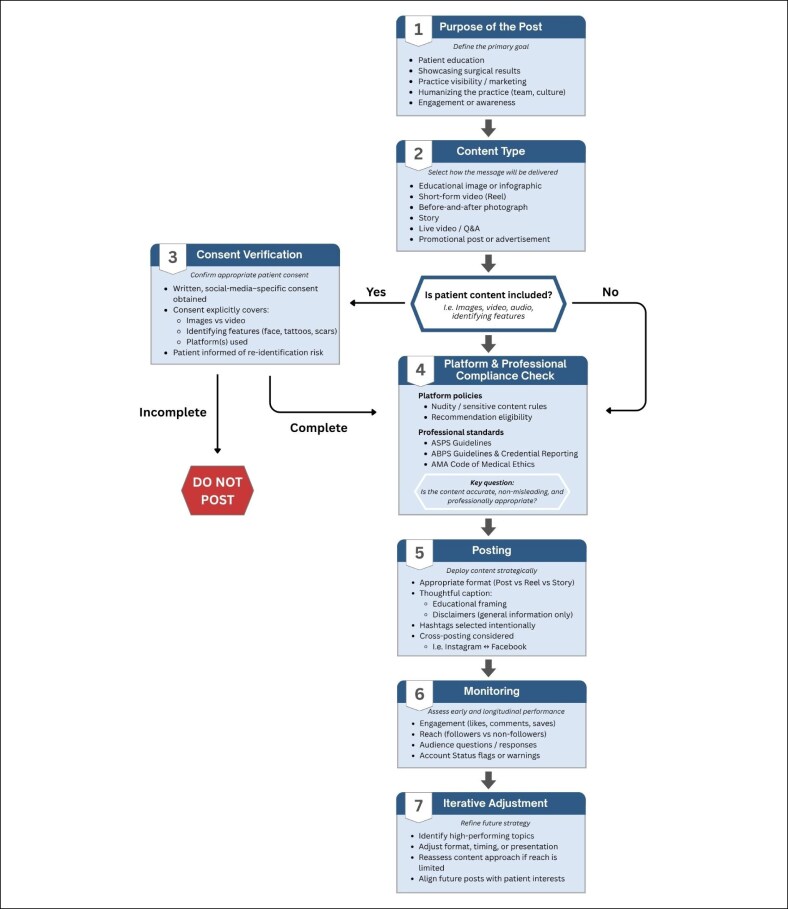
Instagram posting workflow for plastic surgeons. Stepwise framework guiding plastic surgeons through Instagram content creation, consent verification, compliance review, posting, and iterative performance monitoring to ensure ethical, educational, and strategically effective use of the platform. ASPS, American Society of Plsatic Surgeons. ABPS, American Board of Plastic Surgeons. AMA, American Medical Association.

For plastic surgeons aiming to optimize visibility and engagement, consistency of posting has been demonstrated to significantly outweigh volume of posting.^29^ Posting regularly—about 3 to 5 times per week—has been found to be effective for maintaining engagement without audience fatigue.^2,29^ Optimal engagement has been observed with posts published on Fridays around 5:00 Pm, likely reflecting increased leisure-time browsing.^30,31^ Surgeons using Business or Creator accounts can further refine their posting schedules via Instagram Insights to pinpoint audience-specific peak activity. Alternatively, third-party applications, such as Buffer, can assess follower patterns and tailor a strategy for optimal posting times.^13^ Refining a consistent workflow for employing such effective strategies and ethical practices, as outlined in Figure 1, is paramount not only to optimizing reach but also to seamlessly integrating the framework into realistic practice.

### Hashtag Strategy

Hashtags may amplify post visibility and enhance discoverability when used strategically. Procedure-specific and location-based hashtags (ie, #PlasticSurgery, #Rhinoplasty, and #NYCPlasticSurgeon) improve reach and engagement by connecting posts to users actively searching related content.^4^ Excessive or irrelevant hashtags may, however, reduce performance. Focused, moderate use yields optimal results.^4^

## ETHICAL AND LEGAL CONSIDERATIONS

The use of social media in a medical context introduces critical ethical and legal responsibilities related to patient privacy, consent, and professional conduct.

### Patient Consent and Privacy

Surgeons should obtain written, social-media specific consent from patients prior to posting any patient images or videos.^32^ Verbal agreement alone is inadequate and fails to fulfill legal or ethical requirements for social media utilization.^33^ Consent forms must precisely detail what will be shared (eg, photographs, videos), on which platforms, and for what purposes (eg, education, marketing, etc.).^33^ Patients should have the ability and autonomy to choose what they feel comfortable disclosing and retain the right to withdraw their consent at any time, knowing that once shared, material may be hard or impossible to entirely erase from the internet.^33^ Consent for social media usage must be acquired separately from consent for surgical or procedural actions to prevent any perception of coercion.^22,32,33^ Patients should be explicitly informed that declining to give consent will not influence their medical treatment or their relationship with the surgeon.^22,32,33^

Furthermore, every image should be thoroughly examined to assess for potentially identifying elements, such as faces, tattoos, birthmarks, or other distinct physical traits.^22,27,33^ When such features are visible, patient consent should explicitly permit their inclusion. Background elements (ie, facility names, personnel, or equipment) and metadata (ie, Exif data from digital cameras) should also be reviewed, as they may unintentionally disclose patient identity.^22,27,33^ If surgeons elect to blur or otherwise obscure identifying characteristics, patients should be informed that de-identification does not guarantee anonymity, as individuals may remain identifiable based on other unique features, contextual details, or the timing of posts.^22,27,33^

### Professional Responsibility

Physicians should avoid establishing formal doctor–patient relationships through social media, in accordance with guidance from the American Medical Association (AMA) and state medical boards. While platforms such as Instagram may appropriately be used to communicate a surgeon's expertise, scope of practice, and available procedures, and may assist patients in deciding whether to pursue an in-person consultation, social media should not function as a mechanism for initiating or delivering medical care. This distinction is essential to preserve professional boundaries, protect patient privacy, and maintain compliance with regulatory standards.

Although comments and direct messages allow for meaningful engagement, surgeons must remain vigilant that such interactions are not misconstrued as individualized medical advice.^34^ Social media communication should never replace a comprehensive clinical evaluation conducted in a formal care setting.^34^ When responding to inquiries that reference specific symptoms, diagnoses, or treatment decisions, surgeons should clearly state that responses are for general educational purposes only and do not constitute a medical consultation and that patient-specific recommendations require in-person assessment.^34,35^ Consistent use of such disclaimers helps reinforce appropriate expectations and mitigate legal and professional risk.

Compounding these concerns is evidence that nearly one-third (28%) of individuals presenting as “plastic surgeons” on Instagram are not board-certified by the ABPS. This underscores the responsibility of board-certified plastic surgeons to model transparency, professionalism, and ethical conduct in online spaces where patients increasingly seek medical information.

### Managing Patient Expectations

A critical ethical challenge in using Instagram within plastic surgery is managing the risk of contributing to unrealistic beauty standards.^36,37^ Before-and-after images and curated posts frequently showcase unrealistic standards, occasionally through edited or selectively displayed photos, potentially leading to false expectations and adversely affecting self-esteem and body image.^36,37^ This effect appears particularly pronounced among young women and frequent social media users, with multiple studies demonstrating an association between extensive Instagram use and unfavorable body evaluations and higher rates of body dysmorphic disorder.^36,37^

However, the use of social media for education and awareness has simultaneously empowered patients, which may reflect improved patient satisfaction, greater autonomy, and enhanced patient decision-making capacity.^38^ This duality underscores the importance of responsible content creation. Surgeons should consistently emphasize the realities of surgery, including variability in outcomes, the healing and recovery process, and patient safety considerations, rather than presenting idealized results alone.^38^ By prioritizing education and transparency, surgeons can help align patient expectations with clinical reality while mitigating potential psychosocial harm.

## MEASURING SUCCESS

Quantifying the value of social media in direct financial terms can be challenging, but parallel measures of return on investment (ROI) provide a useful framework for tracking the impact of Instagram on practice growth and brand development. In plastic surgery, social media value is often reflected indirectly through increased visibility, patient inquiries, and consultation volume rather than immediate revenue attribution. In this context, one recent study demonstrated that Instagram ROI increased substantially over time, while early trends were better characterized by exponential growth.^39^ When evaluated longitudinally, these indicators may serve as meaningful endpoints for assessing Instagram's contribution to practice development in plastic surgery.

## CONCLUSIONS

Instagram represents an invaluable platform for plastic surgeons, seamlessly integrating patient education, targeted marketing, authentic engagement, and data-driven practice growth. When leveraged strategically and approached with professionalism, transparency, and patient safety in mind, the platform allows surgeons to amplify visibility, foster patient trust, and deliver accurate, expectation-aligned information. In this context, Instagram functions not simply as a marketing tool, but as a practical extension of modern plastic surgery practice that supports informed decision-making and sustainable practice growth.
